# Sex-specific vulnerabilities in early human neurodevelopment following SARS-CoV-2-induced maternal immune activation

**DOI:** 10.1007/s00787-025-02837-z

**Published:** 2025-08-27

**Authors:** Alexandre Díaz-Pons, Sergio Castaño-Castaño, Víctor Ortiz-García de la Foz, Rosa Ayesa-Arriola

**Affiliations:** 1 Departamento de Investigación en Enfermedades Mentales, Instituto de Investigación Marqués de Valdecilla (IDIVAL), Santander, 39011 España; 2 Escuela de Doctorado de la Universidad de Cantabria (EDUC), Universidad de Cantabria (UC), Santander, 39005 España; 3 Departamento de Medicina y Ciencias de la Salud, Facultad de Medicina, Universidad de Cantabria (UC), Santander, 39011 España; 4 Facultad de Psicología, Universidad Nacional de Educación a Distancia (UNED), Madrid, 28015 España; 5 Departamento de Psicobiología, Facultad de Psicología, Universidad de Oviedo (UO), 33003 Oviedo, España; 6 Instituto de Neurociencias del Principado de Asturias (INEUROPA), 33003 Oviedo, España; 7 Instituto de Investigación Sanitaria del Principado de Asturias (ISPA), 33011 Oviedo, España; 8 Centro de Investigación Biomédica en Red de Salud Mental (CIBERSAM), Instituto de Salud Carlos III, Madrid, 28029 España

**Keywords:** Maternal immune activation (MIA), SARS-CoV-2, Cytokines, Sex vulnerability, Neurodevelopment, Neonatal behavioural assessment scale (NBAS)

## Abstract

This study examines the effects of SARS-CoV-2-induced maternal immune activation (MIA) on early neurodevelopment, focusing on sex-specific vulnerabilities related to early behavioral and regulatory functions, which may be precursors to later developmental or cognitive challenges. A total of 107 mother-infant dyads from the COGESTCOV-19 study were analyzed to assess neurodevelopmental outcomes in male and female infants at six weeks using the Neonatal Behavioral Assessment Scale (NBAS). Maternal and newborn cytokine levels—specifically interleukin-6 (IL-6), interleukin-10 (IL-10), and the IL-6/IL-10 ratio—were measured at the first prenatal visit at the time of study enrolment and at birth to evaluate inflammatory responses and homeostatic balance. Neither maternal nor newborn cytokine levels differed significantly between cases and controls. Significant sex-specific differences were observed in neurodevelopmental outcomes related to maternal SARS-CoV-2 exposure. Female infants exposed in utero showed significantly reduced performance in orientation and state regulation measures compared to exposed male infants and unexposed male and female controls. The findings suggest that SARS-CoV-2-induced MIA — not limited to IL-6/IL-10 balance — may have a differential impact on early neurodevelopment based on sex, underscoring the necessity for targeted interventions to mitigate these effects. Future studies should explore the mechanisms underlying these sex-specific differences and their long-term implications for neurodevelopment.

**Supplementary Information:**

The online version contains supplementary material available at 10.1007/s00787-025-02837-z.

## Introduction

The COVID-19 pandemic, caused by the SARS-CoV-2 virus, has raised serious concerns regarding its impact on vulnerable populations [1]. Pregnant women, due to immune system alterations during gestation, are particularly susceptible to infections, which may pose risks to fetal development [2–4]. Despite vertical transmission of SARS-CoV-2 to the fetus being uncommon, maternal immune activation (MIA) in response to infection has emerged as a significant factor that can also interfere with brain development and potentially result in lasting neurodevelopmental and psychiatric conditions [5–8]. Insights from other viral infections, such as Zika and cytomegalovirus, further illustrate how infections can impact fetal neurodevelopment through diverse pathways and outcomes [9, 10].

Central to the maternal immune environmental response are plasma cytokines such as interleukin-6 (IL-6) and interleukin-10 (IL-10), which represent opposing yet functionally interdependent arms of the inflammatory response. These cytokines play a pivotal role in regulating immune activity during pregnancy and infections, including SARS-CoV-2 [11–13]. IL-6, a pleiotropic pro-inflammatory cytokine, initiates acute phase responses, promotes Th17 differentiation, and can trigger neuroinflammatory cascades that interfere with critical processes such as synaptogenesis and cortical development [7, 14, 15]. Elevated plasma levels of IL-6 have been frequently associated with adverse neurodevelopmental outcomes, such as increased risks for autism (ASD) and schizophrenia spectrum disorders (SSD) [13, 16–19]. However, recent studies have shown that these neural and behavioral effects—such as altered synapse formation, changes in cortical and hippocampal development, and modified social, cognitive, and sensorimotor behaviors—are not uniformly negative, as some outcomes may depend on developmental timing, brain region specificity, compensatory mechanisms, and individual biological variability [8, 10, 20]. Spann et al. (2021) observed that maternal IL-6 concentrations were linked to enhanced cognitive outcomes in children at 14 months, suggesting that neurodevelopmental responses to inflammation may vary across contexts [21]. Conversely, IL-10 acts as a major anti-inflammatory regulator, mitigating the harmful effects of excessive inflammation while also contributing to neurodevelopment by modulating microglial activity and synaptic pruning [22–24]. However, this regulatory axis is bidirectional: excessive anti-inflammatory signaling may suppress essential neuroimmune interactions required for proper brain maturation [25, 26]. The IL-6/IL-10 ratio has thus emerged as a meaningful indicator of immune homeostasis, reflecting shifts in the inflammatory balance that may impact fetal brain development. Its demonstrated utility in stratifying disease severity during SARS-CoV-2 infection further supports its relevance in contexts such as the present study, where maternal infection may disrupt immunological equilibrium [11, 27, 28]. Animal models have elucidated key mechanisms by which MIA disrupts neurodevelopment, showing promising translational potential [29, 30]. Yet, these models often lack the complexity of human pregnancy and disorders, as well as the dynamic nature of viral infections [10, 29, 31–33]. While controlled experimental conditions using polyinosinic-polycytidylic acid and lipopolysaccharide to induce MIA in animals help study discrete cytokine elevations, they may oversimplify the complex, multi-mediator immune responses triggered by real-life infections [33]. This highlights the need for more human cohort studies to better understand the effects of MIA on early neurodevelopment.

Research into MIA’s effects on neurodevelopment has primarily focused on two critical factors: the timing of the infection during pregnancy and the sex of the fetus [10, 34, 35]. These factors have produced mixed insights into how the maternal immune response during gestation affects neurodevelopment [36]. Males have traditionally been considered more vulnerable to prenatal immune activation, given the higher incidence of neurodevelopmental disorders (NDD) such as ASD and attention deficit hyperactivity disorder (ADHD) in boys [37–39]. Bale et al., (2016) suggested that sex differences in placental responses to early prenatal stress – specifically, differences in transcriptional repression and regulation – may contribute to this increased male susceptibility [37]. Due to ethical standards, most causal MIA studies have been conducted using animal models, which often rely on male subjects and genetically homogeneous strains, optimizing cost-efficiency and experimental control but limiting generalizability to females, particularly human females [10, 33, 36, 40–42]. Furthermore, these studies often focus on abnormalities found in adolescent and adult stages while neonatal period has been under investigated, leaving early developmental impacts underexplored [34]. Emerging evidence suggests that female fetuses may also be susceptible to MIA, though their neurodevelopmental disruptions may be subtler and differ, sometimes contrasting, with those observed in males [34, 43]. These disruptions can manifest in areas such as attention, sensory processing, and emotional regulation—domains that, due to their often-subtle presentation compared to more overt symptomatology and consequent lack of access to early intervention, can be overlooked in early childhood but may carry long-term implications [34].

Given the timeframe since the onset of the 2020 pandemic, research on early neurodevelopment and prenatal SARS-CoV-2 remains preliminary, as the oldest children affected are no older than five years by 2025. Early studies have begun to reveal the potential impact of SARS-CoV-2-induced MIA on human development; however, the evidence remains mixed and difficult to interpret. Several studies have reported associations between prenatal SARS-CoV-2 exposure and adverse neurodevelopmental outcomes. For example, Edlow et al. (2022) found that maternal SARS-CoV-2 infection during pregnancy was associated with increased odds of neurodevelopmental diagnoses at 12 months, particularly with third-trimester exposure, and these associations persisted after adjusting for key covariates [44]. A follow-up study (Edlow et al., 2023) identified sex-specific differences, with male offspring showing elevated risk at 12 months but not females; the effects attenuated by 18 months [45]. Similarly, Shah et al. (2023) reported that over half of full-term infants exposed in utero showed developmental vulnerabilities in at least one domain by 16–18 months [46]. Garrido-Torres et al. (2024) further noted a significant association between severe maternal infection and personal-social delays at 12 months, independent of infection timing and maternal stress [47]. In contrast, other studies have reported more favorable or nuanced outcomes. Ayed et al. (2022) observed that 90% of infants born to infected mothers had typical development, though first- and second-trimester exposures carried higher risks of delay [48]. Firestein et al. (2023) observed no developmental differences in infants exposed to asymptomatic or mild maternal infections [49]. Population-level studies examining broader pandemic-related factors yielded similarly mixed results. Huang et al. (2021) reported increased risk of fine motor and communication delays at 12 months among pandemic-born firstborns [50]. Apa et al. (2024) found mostly normal auditory and communicative outcomes in children that were prenatally exposed to SARS-CoV-2 [51]. Meta-analyses provide additional context: while Hessami et al. (2022) concluded that gestational SARS-CoV-2 exposure was not consistently associated with neurodevelopmental impairment, they identified elevated risks of fine motor and communication delays; Pinheiro et al. (2023) similarly found no increased delay prevalence but noted lower scores in fine motor and problem-solving domains compared to controls [52, 53]. Former insights from our own cohort warrant mention. Ayesa-Arriola et al. (2023) identified reduced social interaction and motor development following third-trimester SARS-CoV-2 exposure in a pilot sample, while Díaz-Pons et al. (2025) reported few trimester-specific alterations in motor skills, reflexes, and state regulation that correlated with cytokine profiles at complete 6-week follow-up [28, 54]. Summarising, although some studies find no significant neurodevelopmental differences and others report subtle, domain-specific effects associated with prenatal exposure to SARS-CoV-2, there is a consensus on the critical need for longitudinal monitoring to accurately characterize developmental trajectories.

Characterizing these trajectories is essential to understanding how maternal immune response to SARS-CoV-2 during pregnancy—and potential sex-specific differences—may influence early neurodevelopment. This knowledge is particularly critical in the context of the recent pandemic and for anticipating hypothetical future maternal–fetal health challenges. This study seeks to address these gaps by examining the neurodevelopmental outcomes of infants born to mothers infected with SARS-CoV-2, compared to unexposed controls, investigating the infant’s sex as a moderating factor, and expanding upon our previous work on this cohort that focused on the consequences of timing of exposure at the same stage as the present study [28]. To this end, our study assessed early neurodevelopmental outcomes and primitive reflexes as early as six weeks of age using the Neonatal Behavioral Assessment Scale (NBAS) [55], a time point that precedes many postnatal environmental influences, thus minimizing confounding factors [56]. This early evaluation strengthens our capacity to detect subtle effects of prenatal exposure and to trace their evolution over time. We hypothesize that maternal exposure to SARS-CoV-2 during pregnancy will induce distinct inflammatory responses characterized by elevated cytokine concentrations and an increased IL-6/IL-10 ratio, reflecting a pro-inflammatory imbalance. These immune alterations are expected to adversely impact neurodevelopment, with potential sex-specific effects.

## Methods

### Sample

This study analyzed data from 107 mother-infant dyads in the COGESTCOV-19 study conducted in Cantabria, Spain, from December 2020 to February 2022 [54]. The cohort included 59 SARS-CoV-2-exposed mothers (cases) and 48 unexposed mothers (controls), matched by maternal age, parity, and estimated delivery date, with all pregnancies being naturally conceived. Nine participants initially enrolled as controls were reclassified as cases upon subsequent SARS-CoV-2 infection during pregnancy, prompting repetition of all baseline assessments, including blood sampling. Group sizes reflect this final classification. The groups were stratified by infant sex, comprising 36 males and 23 females in the case group, and 25 males and 23 females in the control group, referred to as case male, case female, control male, and control female. Participant recruitment was facilitated through the voluntary collaboration of midwives and obstetricians from the Marqués de Valdecilla University Hospital and various primary care centres across Cantabria, who informed eligible pregnant women about the study. Simultaneously, additional outreach was conducted via social media platforms. Infected status was confirmed through at-clinic PCR testing, while control mothers were assigned based on self-report and absence of symptoms. All participants provided written informed consent. The study was approved by the Marqués de Valdecilla University Hospital Review Board (approval code 2020.190) and complied with the 1964 Declaration of Helsinki.

### Interviews and data collection

Data were collected through comprehensive semi-structured interviews conducted both prenatally and postnatally. Prenatal interviews collected sociodemographic data and assessed psychological status using the following instruments: the State-Trait Anxiety Inventory (STAI) [57], the Prenatal Distress Questionnaire (PDQ) [58], the Social Readjustment Rating Scale (SRSS) [59], the Couple Relationship Quality Scale (CRP) [60], the Oviedo Sleep Questionnaire (COS) [61], Perceived Social Stress (PSS) [62] and the Fear of COVID-19 Scale [63]. Cases were evaluated for symptom presence (yes/no)—including fever, cough, fatigue, myalgia, diarrhea, headache, and others—and infection severity was categorized according to previously established criteria (hospitalized vs. home convalescence) [64]. Postnatal interviews included questions about delivery outcomes and neonatal health indicators, such as APGAR scores at 1 and 5 min and postnatal depression was assessed using the Edinburgh Postnatal Depression Scale (EPDS) [65]. Interviews were blindly conducted by trained psychologists to ensure consistency and reliability. Further methodological details can be found in Barrio-Martinez et al., 2024 [66].

### Neurodevelopmental assessment

Neurodevelopmental outcomes at six weeks of age were assessed using the Neonatal Behavioral Assessment Scale (NBAS) [55]. The NBAS evaluates 27 behavioral items across six subdomains: Habituation, Social-Interactive/Orientation, Motor System, State Organization, State Regulation, and Autonomic Stability. To address the variability in item completion—particularly for those requiring specific infant states (e.g., habituation during sleep or orientation during alertness)—composite scores were calculated following previous methodology using two approaches [28]. Selective composite scores were based on participants with complete data across all items, ensuring equal item weighting but reducing sample size. Comprehensive composite scores included all available data, maximizing sample size but potentially introducing bias due to unequal item representation. Additionally, 18 primitive reflexes (Babinski, ankle clonus, rooting, sucking, glabella, passive movements of the legs and arms, palmar grasp, placing, standing, walking, crawling, incurvation, tonic deviation of the head and eyes, nystagmus, tonic neck reflex, and Moro reflex) were assessed to gauge central nervous system integrity. All assessments were conducted in a controlled laboratory environment at the Instituto de Investigación Marqués de Valdecilla (IDIVAL), with evaluations blindly performed by a certified and independent examiner to minimize bias.

### Cytokines

Maternal blood samples were collected during the first prenatal visit at the time of study enrolment (week range: 8–40), and cord blood samples were obtained at birth. All samples were immediately stored at −80 °C in the Valdecilla Biobank. Plasma was extracted by centrifugation at 3000 rpm for 10 min at room temperature. Interleukin-6 (IL-6) and Interleukin-10 (IL-10) concentrations were measured using Enzyme-Linked Immunosorbent Assay (ELISA) kits from Enzo Life Sciences: the high sensitivity ELISA Kit (Catalog #: ENZ-KIT178-0001) for IL-6 and the high sensitivity ELISA Kit (Catalog #: ADI-900-036) for IL-10, following the manufacturer’s instructions. Data were log-transformed to normalize the distribution to minimize data dispersion and reduce potential biases. Further details in Díaz-Pons et al., (2025) [28].

### Statistical analysis

Data were analyzed using R version 4.3.2 (2024-05-27). Sociodemographic, clinical, and physiological variables were compared using chi-square or Fisher’s exact tests for categorical data, and t-tests, Mann-Whitney U tests, or ANOVAs for continuous variables, as appropriate (see Tables S1 and S2). Primary analyses focused on neurodevelopmental outcomes and were conducted using ANCOVAs to examine infection status × sex interactions (Table 1). Models adjusted for covariates with established relevance to early neurodevelopment—maternal age, maternal education, gestational age at birth, infant age at NBAS assessment, and infant weight—and accounted for group differences with potential environmental influence on neurodevelopment by including maternal annual salary. Bonferroni correction was applied to post hoc comparisons. All statistical tests were two-tailed, with significance set at *p* < 0.05.

**Table 1:** Significant NBAS scores of COGESTCOV19 newborns at 6-week follow-up: case-control by sex subdivision

	Case Male			Case Female			Control Male			Control Female			Adjusted for: Mother’s age, gestational age, infant’s age at NBAS assessment, years of education, child’s weight and height, salary				
	*N* = 32			*N* = 22			*N* = 24			*N* = 22							
	*n*	Mean	SD	*n*	Mean	SD	*n*	Mean	SD	*n*	Mean	SD	Statistical (df)	Value	*P*-value	Effect Size (η²)	Post-Hoc
Orientation																	
6-Animate visual and auditory orientation	30	6.5	2.3	20	4.3	2.6	18	6.5	2.7	21	6.5	2.6	F (3. 85)	4.132	0.009	0.137	1 > 2 **
8-Inanimate visual and auditory orientation	28	6.1	2.0	20	3.6	2.1	18	5.6	3.1	19	5.7	2.8	F (3. 81)	4.675	0.005	0.159	1 > 2 **
10-Inanimate auditory orientation	29	6.5	1.8	20	5.2	2.0	19	6.4	1.8	19	5.3	2.1	F (3. 83)	4.108	0.009	0.140	1 > 2 1 > 4 *
11-Alertness	29	6.2	2.3	20	4.4	2.7	19	5.8	2.3	21	5.7	2.6	F (3. 85)	2.683	0.052	0.094	1 > 2 *
Orientation selective composite score	25	5.7	1.8	18	4.2	1.7	16	5.6	1.9	18	5.7	2.2	F (3. 73)	3.130	0.031	0.125	1 > 2 *
Orientation comprehensive composite score	30	5.9	1.7	20	4.0	1.7	20	5.5	1.9	22	5.4	2.2	F (3. 88)	4.815	0.004	0.151	1 > 2 **
Supplementary items																	
30-Cost of attention	32	6.7	2.0	22	5.5	1.8	24	5.6	2.6	22	5.5	2.0	F (3. 96)	4.521	0.005	0.132	1 > 4 **; 1 > 2 *
31-Examiner facilitation	32	6.2	2.4	22	5.3	1.5	24	5.3	2.6	22	5.0	2.1	F (3. 96)	3.997	0.010	0.119	1 > 4 **
33-Robustness and endurance	32	6.8	2.3	22	6.0	2.0	24	5.8	2.6	22	5.6	2.9	F (3. 96)	3.087	0.031	0.094	1 > 4 *
34-State regulation	32	7.8	1.4	22	6.5	2.1	24	6.8	2.4	22	7.7	1.5	F (3. 96)	4.148	0.008	0.123	1 > 2 1 > 3 *
Reflexes																	
3-Ankle clonus	31	1.5	0.5	20	1.4	0.5	23	1.5	0.5	20	2.0	0.2	F (3. 93)	5.763	0.001	0.172	2 < 4 3 < 4 **; 1 < 4 *
14-Incurvation (galiant response)	31	1.8	0.4	22	1.6	0.5	21	1.7	0.5	22	2.0	0.0	F (3. 96)	3.166	0.029	0.101	2 < 4 *

** *p* < 0.01; * *p* < 0.05

## Results

### Sociodemographic, clinical, and physiological findings

**Fig. 1:**
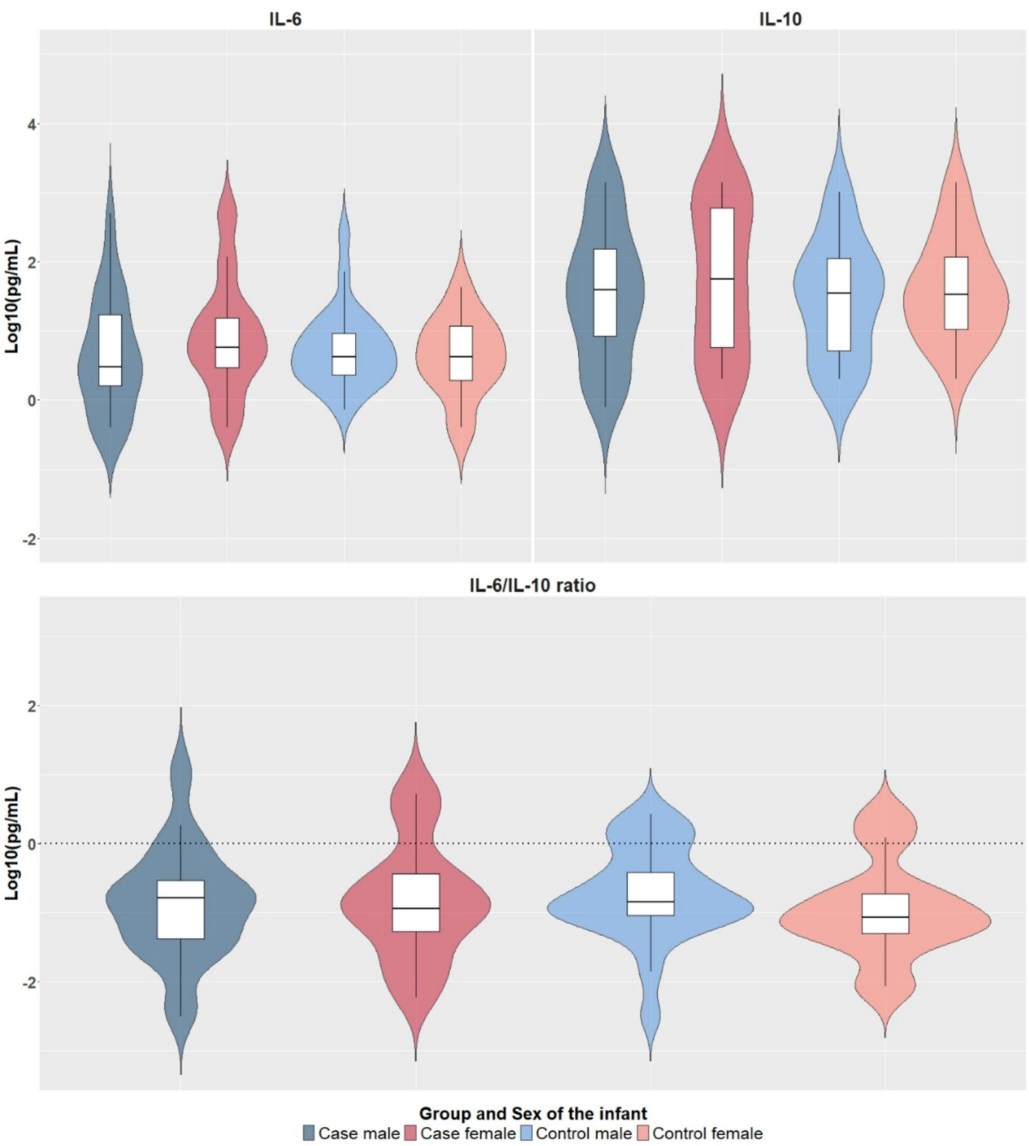
The top panels show log₁₀-normalized IL-6 and IL-10 levels measured in maternal blood during pregnancy for cases and controls, stratified by sex. The bottom panel displays the corresponding IL-6/IL-10 ratios. A log₁₀ IL-6/IL-10 value of 0 corresponds to a raw ratio of 1 (log₁₀(1) = 0), indicating equilibrium between IL-6 and IL-10 (dashed line). Positive values indicate a pro-inflammatory state (IL-6 > IL-10), while negative values reflect an anti-inflammatory state (IL-6 < IL-10). No significant group differences were observed in any of the three measures

No significant differences were observed between exposed and control groups in maternal age, parity, delivery date, education, marital status, gestational age, APGAR scores, or trimester of infection among cases (all *p* > 0.05). Mothers of female cases had lower annual incomes compared to control females (χ² = 14.39, *p* ≤ 0.002, V = 0.364). Female case infants were shorter than male controls (F = 5.44, *p* ≤ 0.002, η² = 0.40). No differences were found in maternal IL-6, IL-10, and IL-6/IL-10 ratio between cases and controls, stratified by the sex of their infant (Fig. 1). Similarly, no differences were found in newborn IL-6, IL-10, and IL-6/IL-10 ratio between groups. Table S1 summarizes the characteristics of the mother-newborn dyads.

### Neurodevelopmental outcomes by sex and infection status findings

**Fig. 2:**
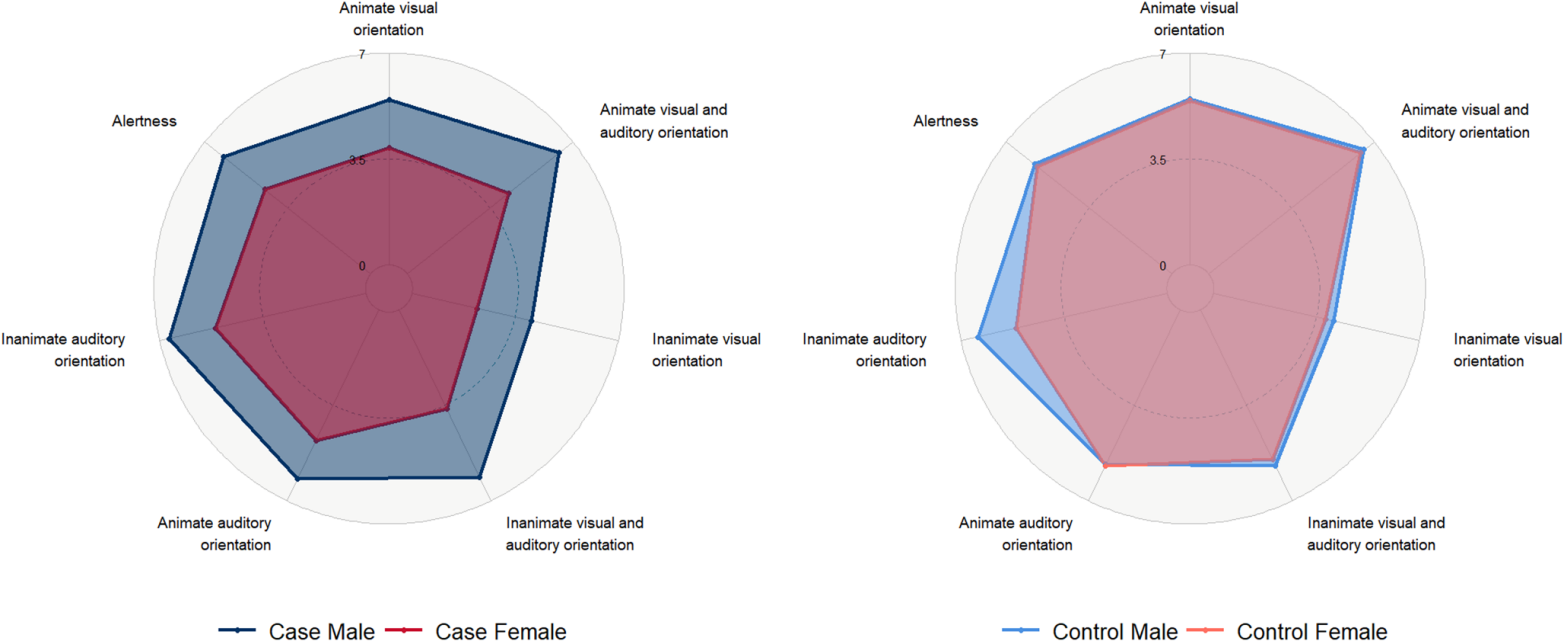
Sex-stratified case-control comparisons in the NBAS orientation domain are reported as mean scores: female cases consistently underperformed male cases, with statistically significant differences in animate visual and auditory orientation, inanimate visual and auditory orientation, inanimate auditory orientation, and alertness

Female cases showed poorer performance than male cases on Animate Visual and Auditory Orientation (F = 4.13, *p* ≤ 0.01, η² = 0.14), Inanimate Visual and Auditory Orientation (F = 4.68, *p* ≤ 0.01, η² = 0.16), and Inanimate Auditory Orientation (F = 4.11, *p* ≤ 0.01, η² = 0.14) (Fig. 2). Furthermore, female cases showed significantly poorer performance than male cases on both the Orientation Selective (F = 3.13, *p* ≤ 0.05, η² = 0.12) and Comprehensive Composite Scores (F = 4.82, *p* ≤ 0.01, η² = 0.15). Female cases underperformed compared to male cases in State Regulation (F = 4.15, *p* ≤ 0.01, η² = 0.12) and Cost of Attention (F = 4.52, *p* ≤ 0.01, η² = 0.13). Female cases also showed lower scores than female controls in reflexes, including Ankle Clonus (F = 5.76, *p* ≤ 0.001, η² = 0.17) and Incurvation (F = 3.17, *p* ≤ 0.05, η² = 0.10). Male cases exhibited poorer outcomes only in Ankle Clonus compared to female controls (F = 5.76, *p* ≤ 0.001, η² = 0.17).

Female controls performed worse than male cases in Inanimate Auditory Orientation (F = 4.11, *p* ≤ 0.01, η² = 0.14), Cost of Attention (F = 4.52, *p* ≤ 0.01, η² = 0.13), Examiner Facilitation (F = 3.99, *p* ≤ 0.01, η² = 0.12), and Robustness and Endurance (F = 3.09, *p* ≤ 0.05, η² = 0.09). Male controls showed worse performance in State Regulation compared to male cases (F = 4.15, *p* ≤ 0.01, η² = 0.12) and had poorer outcomes in Ankle Clonus compared to female controls (F = 5.76, *p* ≤ 0.001, η² = 0.17). Significant and complete NBAS outcomes at six weeks of age are respectively presented in Table 1 and Table S2.

## Discussion

This study offers novel insights into the sex-specific neurodevelopmental vulnerabilities induced by maternal SARS-CoV-2 infection during pregnancy. Contrary to our hypothesis, maternal cytokine levels did not differ significantly between cases and controls. Female infants showed poorer orientation outcomes on the NBAS despite similar inflammatory exposures to SARS-CoV-2, challenging assumptions of female resilience to prenatal adversity. These significant findings challenge the often-presumed resilience of females to prenatal adversities and suggest that females may also be susceptible to certain neurodevelopmental disruptions following MIA.

### Maternal cytokine ratios and immune balance: beyond a simple inflammatory marker

The immune response observed during real-world infections, such as SARS-CoV-2, is far more complex than the experimental cytokine manipulations typically studied in animal models. In real infections, multiple cytokines interact in dynamic networks, individual variability shapes immune responses, and the course of infection evolves over time, factors that are often absent in controlled animal studies. Our study emphasizes the importance of analyzing the balance between pro-inflammatory IL-6 and anti-inflammatory IL-10 markers rather than focusing solely on isolated cytokine levels. This balance provides a more nuanced understanding of how immune regulation may impact neurodevelopment [12, 27]. Interestingly, no statistically significant differences in IL-6/IL-10 ratios were found. All study groups exhibited mean ratios below 0 on the logarithmic scale, indicating that anti-inflammatory agent IL-10, played a dominant role in controlling IL-6 inflammation. Nonetheless, visual inspection of the plots suggested key patterns.

Control group participants, particularly mothers of females, exhibited lower and more consistent IL-6/IL-10 ratios, suggesting a more stable and controlled inflammatory balance, which might come as a logical consequence of the nature of this group. In contrast, the case group—especially among mothers of female participants—demonstrated greater variability in these ratios, with slightly elevated mean values. This variability suggests a heightened and less regulated inflammatory response in individuals with SARS-CoV-2 infection. In line with findings from previous SARS-CoV-2 research, the absence of statistical differences across the groups could be attributed to the mild nature of infections within our cohort as only one woman required hospitalization, with the remaining participants recovering at home [64, 66–68]. Importantly, clinical records indicated that none of the 107 women in this cohort received antiviral treatment during pregnancy, allowing us to reasonably exclude direct pharmacological effects as a contributing factor to our MIA measurements. Nonetheless, other sociocultural mediators warrant consideration in cross-cultural research. Beyond the role of Spain’s universally accessible public healthcare system—which ensures early diagnosis, specialist follow-up, and comprehensive care at no cost—factors such as general health literacy, culturally embedded health-seeking behaviors, and access to reliable medical information may have also contributed to attenuated immune responses and reduced variability across groups [10, 69]. However, this remains a hypothesis that warrants further investigation, particularly in resource-limited settings where disparities in care and public health infrastructure may shape both the extent of maternal immune activation and the ability to monitor its neurodevelopmental consequences [69]. Notably, mothers of females in the exposed group actually had slightly higher IL-6/IL-10 ratios compared to mothers of males (− 0.8 vs. −0.7), reflecting greater proinflammatory imbalance and might be relevant to explain the neurodevelopmental differences found.

### Sex-specific vulnerabilities: breaking conventional norms

Sex-specific vulnerabilities in NDD may have been historically underestimated, partly due to a male bias in research and clinical focus [38, 70]. Males often exhibit more visible externalizing behaviors, such as hyperactivity and aggression, which are easier to identify in educational and clinical settings [71, 72]. In contrast, females frequently present subtler signs, including attention deficits and sensory processing issues, which may be more difficult to detect, especially in early childhood [72–77]. This diagnostic discrepancy might have contributed to the possible misconception that females are less prone to NDD [78–80]. However, emerging research, including our own findings, suggests that females may be equally, or in some cases more, vulnerable to certain neurodevelopmental challenges, though these may manifest differently from those typically observed in males [40, 43].

In our study, only one significant sex difference was noted among the control group. Remarkably, despite similar prenatal inflammatory conditions among cases, female infants appeared to experience greater difficulties in neurobehavioral areas such as orientation, state regulation, and, to a lesser extent, alertness (the latter showing a trend toward significance; *p* = 0.052). These neurobehavioral domains assessed in early infancy capture foundational aspects of sensory-attentional processing, motor control, and self-regulation, which are critical for later cognitive and emotional development. The orientation domain, reflecting an infant’s capacity to visually and auditorily engage with stimuli, has been linked to the early development of social cognition and attentional systems [81, 82]. Regulation of behavioral states—such as transitions between alertness, sleep, and distress—is essential for effective environmental interaction and emotional adaptability, and difficulties in this domain have been associated with later behavioral and emotional dysregulation [83]. Cost of attention, conceptualized as the infant’s capacity to maintain optimal engagement in the presence of fatigue or competing stimuli, has been identified as a potential early marker of emerging attentional vulnerabilities, including risk for later ADHD [84]. Reflexive motor responses such as ankle clonus and incurvation serve as proxies of neurodevelopmental maturity, where exaggerated or poorly modulated responses may reflect immature or dysregulated neural circuits [85]. However, these reflexes typically integrate by 4–6 months; although female controls outperformed female cases (and males in incurvation), these differences at the 6-week assessment remain clinically nonsignificant but may hold relevance for future developmental outcomes. Finally, examiner facilitation and robustness/endurance—indices of the infant’s ability to sustain performance and modulate arousal—offer insight into the developing self-regulatory systems that support the emergence of executive function [86]. Collectively, these domains provide a window into the early neurodevelopmental processes that underlie later functional outcomes and may serve as sensitive indicators of subtle disruptions following prenatal adversity. Given this, we call for cautious interpretation and emphasize that these should be considered exploratory findings, which do not irrefutably imply causality. It is important to differentiate broader functional domain differences (e.g., orientation) from isolated findings (e.g., ankle clonus) that may reflect incidental results. We recommend cross-cohort replication to clarify whether these effects are cohort-specific, transient, compensatory, or early indicators of long-term neurodevelopmental risk. Their clinical significance requires confirmation through longitudinal follow-up.

Previous research has linked early-life behaviors, such as visual and auditory disengagement of attention—the processes by which infants shift their focus from one visual or auditory stimulus to another—to an increased risk of developing ASD in later years, albeit not specifically in females [87–92]. Similarly, our findings are in line with previous research suggesting that females may exhibit early sexually dimorphic symptoms in more subtle forms—such as anxiety, social withdrawal, or inattentiveness—compared to the more overt and externally visible behaviors commonly observed in males, such as pronounced repetitive behaviors in ASD or hyperactivity in ADHD [93]. While classically male-associated symptoms like repetitive behaviors can also occur in females, they often present in more internalized or socially acceptable forms, such as intense, focused interests or repetitive thought patterns rather than observable motor behaviors [93]. This subtler presentation —not in terms of magnitude but in the overt detectability of behaviors—may render females “protected” from a formal diagnosis, rather than from the underlying condition itself. Despite these early differences, core features of the disorder often become more apparent over time, particularly as the increasing complexity of social and environmental demands makes compensatory or “camouflaging” strategies—such as mimicking facial expressions, maintaining eye contact, or rehearsing social scripts—progressively harder to sustain [75, 94]. Moreover, recent studies, including Guma & Chakravarty (2025), have reported similar sex-specific patterns, particularly in the context of MIA [10, 95, 96]. In addition, Simanaviciute et al. (2024) recently reported that female offspring of MIA rats exhibited reduced whisker angles during object exploration, indicating a possible attentional deficit in these females [97]. The early emergence of these signs could indicate the need for more tailored screening methods to capture neurodevelopmental risks in females, which might otherwise be overlooked under traditional focus on male-dominated symptoms [39, 78, 98].

Given the challenges in detecting these subtler symptoms, there may be value in considering closer postnatal monitoring for all infants exposed to prenatal immune activation, including those exposed to SARS-CoV-2, with particular attention to female infants. While the subtler nature of female neurodevelopmental symptoms may contribute to underdiagnosis, early detection could help mitigate potential long-term complications [98]. Developing more specific evaluation tools that target neurodevelopmental alterations in females might be a useful step, as current postnatal monitoring tends to emphasize male-typical symptoms [39, 42, 78, 98]. Raising awareness among healthcare providers of these sex differences could improve the accuracy and timeliness of diagnoses in both male and female infants [78, 98].

There is also a critical need for longitudinal studies to better understand whether these early neurodevelopmental differences in females persist or evolve over time [6, 35, 47]. Previous research suggests that early neurobehavioral disruptions may be linked to later cognitive difficulties, though these effects may not become apparent until later developmental stages [56]. Long-term follow-up could clarify whether these early signs are predictive of future challenges, helping to inform early intervention strategies [44, 46, 48, 54]. Furthermore, the relatively homogenous nature of many existing studies on prenatal immune activation and neurodevelopment might limit the generalizability of their findings [35]. Expanding research to include more diverse and multicentric cohorts, covering a wider range of geographic, socioeconomic, and genetic contexts, could enhance our understanding of how prenatal exposures, including SARS-CoV-2, influence neurodevelopment across different populations. Such efforts may ultimately support the development of more inclusive and effective interventions tailored to various demographic groups.

### Strengths and limitations

One of the key strengths of this study is its focus on a real-world human cohort, which provides a valuable opportunity to observe the effects of maternal immune responses on early neurodevelopment in a naturalistic setting. Unlike controlled laboratory experiments, our study reflects the complexities of MIA in response to SARS-CoV-2 infection and its impact on offspring neurodevelopment. The careful matching of participants allowed us to minimize confounding variables, and the use of an extensive tool like NBAS at a very young age such as 6 weeks of age was particularly beneficial in detecting early and subtle sex-specific neurodevelopmental differences. While these differences may seem minor, they could become clinically significant as children grow.

However, our study also has several limitations. The small sample size limits the generalizability of our findings and may have reduced our ability to detect smaller, yet significant, differences between groups. Additionally, while our focus on IL-6 and IL-10 as markers of immune activation provided important insights, other cytokines such as TNF-α and IL-1β, which are also known to play crucial roles in neurodevelopment, were not measured. Notably, while PCR testing at study entry confirmed infection status, post-enrolment and asymptomatic infections in the control group cannot be entirely ruled out, despite proactive reporting from participants motivated by concern for their own and their infants’ health.

### Future directions

Future research should emphasize inclusive studies that address sex-specific neurodevelopmental vulnerabilities in the context of MIA. Identifying critical gestational periods during which MIA differentially affects male and female neurodevelopment is vital for developing targeted interventions. Additionally, refining diagnostic criteria and early intervention protocols to detect subtle impairments, particularly in females, is crucial, as current tools may overlook these signs, leading to delayed or missed diagnoses. Expanding the range of immune markers studied will offer a broader understanding of how maternal immune responses differentially impact male and female neurodevelopment. Advanced neuroimaging and neurophysiological techniques will be essential to reveal the structural and functional brain changes linked to prenatal SARS-CoV-2 exposure, further elucidating the mechanisms behind these sex-specific risks, beyond task performance. Moreover, systemic and sociocultural factors—including maternal healthcare access, health literacy, and public health infrastructure—warrant systematic integration into MIA research, given their potential to shape both its biological expression and neurodevelopmental impact. Finaly, future studies would benefit from longitudinal designs incorporating repeated virological assessments across pregnancy to minimize classification uncertainty related to potential post-enrolment asymptomatic infections.

## Conclusions

Our study indicates that females may be more susceptible to mild SARS-CoV-2-induced MIA than previously recognized. Exposed female infants showed poorer orientation outcomes compared to males, challenging the traditional view of greater male vulnerability to prenatal immune disruptions. These subtler behavioral differences, unlike the more noticeable male-typical externalizing NDD symptoms, may go undetected for longer. Longitudinal studies are essential to capture how early immune challenges shape long-term outcomes, with multiple time-point assessments providing a more complete picture of developmental trajectories to identify potential interventions. Expanding research to diverse populations is crucial for understanding how sex-specific vulnerabilities manifest across different contexts.

## Supplementary Information

Below is the link to the electronic supplementary material.

Supplementary Material 1 (DOCX 45.5 KB)

Supplementary Material 2 (DOCX 46.7 KB)

## Data Availability

The data supporting the findings of this article is available upon request from the corresponding author, RAA.
