# Supplementary material for: Sex-specific vulnerabilities in early human neurodevelopment following SARS-CoV-2-induced maternal immune activation
Source: Eur Child Adolesc Psychiatry. 2025 Aug 27;35(1):239–51. doi: 10.1007/s00787-025-02837-z (PMC12916955; doi:10.1007/s00787-025-02837-z)
Supplement: Supplementary file 1 — (DOCX 45.5 KB) [file 787_2025_2837_MOESM1_ESM.docx]

| **Table S1.** Sociodemographic, clinical, and physiological information of COGESTCOV19 mother-newborn dyads at 6-week follow-up: sex-stratified case-control subdivision. | | | | | | | | | | | | | | | | | | | | | | | | |
| --- | --- | --- | --- | --- | --- | --- | --- | --- | --- | --- | --- | --- | --- | --- | --- | --- | --- | --- | --- | --- | --- | --- | --- | --- |
|  | **Case Male** | | | | **Case Female** | | | | **Control Male** | | | | **Control Female** | | | |  |  |  |  |  | |  |  |
|  | **N= 36** | | | | **N= 23** | | | | **N= 25** | | | | **N= 23** | | | |  |  |  |  |  | |  |  |
|  | **n** | **Mean** | **SD** | **n** | | **Mean** | **SD** | **n** | | **Mean** | **SD** | **n** | | **Mean** | **SD** | **Statistical (*df*)** | | **Value** | ***P*-value** | **Effect size (η²)** | | **Post-Hoc** | |  |
| **Sociodemographic** |  |  |  |  | |  |  |  | |  |  |  | |  |  |  | |  |  |  | |  | |  |
| *Mother* |  |  |  |  | |  |  |  | |  |  |  | |  |  |  | |  |  |  | |  | |  |
| Age (years) | 36 | 34.1 | 4.2 | 23 | | 33.2 | 2.8 | 25 | | 34.2 | 3.4 | 23 | | 34.6 | 4.0 | F (3.103) | | 0.612 | 0.608 |  | |  | |  |
| Years of education | 36 | 15.2 | 4.1 | 23 | | 15.9 | 5.6 | 25 | | 18.2 | 4.0 | 23 | | 17.6 | 4.6 | F (3.103) | | 2.827 | 0.042 | 0.287 | | ns | |  |
|  | **N** | **n** | **(%)** | **N** | | **n** | **(%)** | **N** | | **N** | **(%)** | **N** | | **n** | **(%)** |  | |  |  |  | |  | |  |
| Married or with partner | 36 | 33 | 91.7 | 23 | | 22 | 95.7 | 25 | | 24 | 96.0 | 23 | | 21 | 91.3 | Fisher | | 0.809 | 0.872 |  | |  | |  |
| Anual salary (>€20k) | 33 | 24 | 72.7 | 22 | | 15 | 68.2 | 24 | | 11 | 45.8 | 22 | | 6 | 27.3 | χ2 (3) | | 14.388 | 0.002 | 0.364 | | 1>4 **; 2>4 * | |  |
| Primiparous (Yes) | 36 | 23 | 63.9 | 23 | | 11 | 47.8 | 25 | | 14 | 56.0 | 23 | | 14 | 60.9 | χ2 (3) | | 1.608 | 0.658 |  | |  | |  |
| Alcohol (Yes) | 36 | 1 | 2.8 | 23 | | 2 | 8.7 | 25 | | 1 | 4.0 | 22 | | 2 | 9.1 | Fisher | | 1.571 | 0.722 |  | |  | |  |
| Tobacco (Yes) | 36 | 2 | 5.6 | 23 | | 2 | 8.7 | 25 | | 1 | 4.0 | 23 | | 2 | 8.7 | Fisher | | 0.670 | 0.793 |  | |  | |  |
| Trimester of infection | 36 |  |  | 23 | |  |  |  | |  |  |  | |  |  |  | |  |  |  | |  | |  |
| 1st | - | 6 | 60.0 | - | | 4 | 40.0 | - | | - | - | - | | - | - | Fisher | | 0.005 | 1.000 |  | |  | |  |
| 2nd | - | 16 | 59.26 | - | | 11 | 40.74 | - | | - | - | - | | - | - | Fisher | | 0.065 | 1.000 |  | |  | |  |
| 3rd | - | 14 | 63.64 | - | | 8 | 36.36 | - | | - | - | - | | - | - | Fisher | | 0.101 | 0.789 |  | |  | |  |
| *Newborn* | **n** | **Mean** | **SD** | **n** | | **Mean** | **SD** | **n** | | **Mean** | **SD** | **n** | | **Mean** | **SD** |  | |  |  |  | |  | |  |
| Gestational age (weeks) | 36 | 40.1 | 1.2 | 23 | | 40.0 | 1.2 | 25 | | 40.0 | 1.0 | 23 | | 39.7 | 1.2 | H(3) | | 2.249 | 0.522 |  | |  | |  |
| Age (days) | 36 | 45.2 | 8.4 | 23 | | 43.1 | 2.4 | 25 | | 44.0 | 2.8 | 23 | | 43.4 | 2.5 | H (3) | | 1.416 | 0.702 |  | |  | |  |
| 1st minute APGAR score | 35 | 8.5 | 1.4 | 21 | | 8.7 | 1.0 | 25 | | 8.5 | 0.8 | 21 | | 8.5 | 1.2 | H (3) | | 1.716 | 0.633 |  | |  | |  |
| 5th minute APGAR score | 35 | 9.5 | 0.9 | 21 | | 9.6 | 0.9 | 25 | | 9.6 | 0.6 | 21 | | 9.6 | 0.7 | H (3) | | 1.384 | 0.709 |  | |  | |  |
|  | **N** | **n** | **(%)** | **N** | | **n** | **(%)** | **N** | | **n** | **(%)** | **N** | | **n** | **(%)** |  | |  |  |  | |  | |  |
| Full term (yes) | 36 | 35 | 97.2 | 23 | | 23 | 100.0 | 25 | | 25 | 100.0 | 23 | | 23 | 100.0 | Fisher | | 1.991 | 1.000 |  | |  | |  |
| Natural birth (yes) | 36 | 25 | 69.4 | 23 | | 12 | 52.2 | 25 | | 10 | 40.0 | 23 | | 13 | 59.1 | χ2 (3) | | 5.461 | 0.141 |  | |  | |  |
| **Clinical** | **n** | **Mean** | **SD** | **n** | | **Mean** | **SD** | **n** | | **Mean** | **SD** | **n** | | **Mean** | **SD** |  | |  |  |  | |  | |  |
| PDQ | 36 | 22.8 | 8.3 | 23 | | 19.3 | 7.1 | 25 | | 23.7 | 8.4 | 23 | | 24.5 | 7.7 | F (3.103) | | 1.910 | 0.133 |  | |  | |  |
| SRRS | 36 | 171.9 | 87.9 | 23 | | 127.4 | 59.0 | 25 | | 149.9 | 76.2 | 23 | | 131.8 | 54.2 | F (3.103) | | 2.274 | 0.084 |  | |  | |  |
| CRP Anxiety | 36 | 49.0 | 16.4 | 23 | | 51.0 | 13.9 | 25 | | 52.3 | 14.0 | 23 | | 52.7 | 13.7 | F (3.103) | | 0.373 | 0.773 |  | |  | |  |
| STAI-E | 36 | 71.3 | 5.6 | 23 | | 70.0 | 7.1 | 25 | | 70.2 | 7.1 | 23 | | 67.5 | 7.4 | F (3.103) | | 1.513 | 0.216 |  | |  | |  |
| COS | 36 | 11.4 | 6.3 | 23 | | 10.3 | 6.6 | 25 | | 11.8 | 6.7 | 23 | | 12.5 | 9.4 | F (3.103) | | 0.378 | 0.769 |  | |  | |  |
| PSS | 36 | 32.6 | 8.1 | 23 | | 31.1 | 6.8 | 25 | | 32.6 | 9.6 | 23 | | 35.3 | 8.4 | F (3.103) | | 1.002 | 0.395 |  | |  | |  |
| Fear to COVID-19 scale | 36 | 21.0 | 6.4 | 23 | | 20.8 | 5.8 | 25 | | 21.1 | 8.0 | 23 | | 18.8 | 7.0 | F (3.103) | | 0.624 | 0.601 |  | |  | |  |
| EPDS | 36 | 14.2 | 4.5 | 23 | | 12.5 | 3.8 | 25 | | 14.3 | 6.1 | 23 | | 14.0 | 4.1 | H (3) | | 1.949 | 0.583 |  | |  | |  |
|  | **N** | **n** | **(%)** | **N** | | **n** | **(%)** | **N** | | **N** | **(%)** | **N** | | **n** | **(%)** |  | |  |  |  | |  | |  |
| Hospitalization (yes) | 36 | 1 | 2.8 | 23 | | 0 | 0.0 | - | | - | - | - | | - | - | Fisher | | 1.991 | 1.000 |  | |  | |  |
| Symptoms during COVID-19 (yes) | 35 | 34 | 97.1 | 23 | | 21 | 91.3 | - | | - | - | - | | - | - | Fisher | | 0.965 | 0.556 |  | |  | |  |
| Fever (yes) | 33 | 21 | 63.6 | 22 | | 11 | 50.0 | - | | - | - | - | | - | - | Fisher | | 1.009 | 0.406 |  | |  | |  |
| Cough (yes) | 33 | 21 | 63.6 | 22 | | 11 | 50.0 | - | | - | - | - | | - | - | Fisher | | 1.009 | 0.406 |  | |  | |  |
| Fatigue (yes) | 33 | 14 | 42.4 | 22 | | 11 | 50.0 | - | | - | - | - | | - | - | Fisher | | 0.306 | 0.595 |  | |  | |  |
| Myalgia (yes) | 33 | 16 | 48.5 | 22 | | 12 | 54.5 | - | | - | - | - | | - | - | Fisher | | 0.194 | 0.785 |  | |  | |  |
| Diarrhea (yes) | 33 | 5 | 15.2 | 22 | | 5 | 22.7 | - | | - | - | - | | - | - | Fisher | | 0.509 | 0.498 |  | |  | |  |
| Headache (yes) | 33 | 18 | 54.5 | 21 | | 10 | 47.6 | - | | - | - | - | | - | - | Fisher | | 0.247 | 0.781 |  | |  | |  |
| Others (yes) | 33 | 31 | 93.9 | 22 | | 18 | 81.8 | - | | - | - | - | | - | - | Fisher | | 1.995 | 0.204 |  | |  | |  |
| **Physiological** | 10 | **Mean** | **SD** | **n** | | **Mean** | **SD** | **n** | | **Mean** | **SD** | **n** | | **Mean** | **SD** |  | |  |  |  | |  | |  |
| *Mother* |  |  |  |  | |  |  |  | |  |  |  | |  |  |  | |  |  |  | |  | |  |
| Weight (kg) | 35 | 71.8 | 12.6 | 23 | | 73.1 | 12.2 | 25 | | 72.0 | 13.7 | 23 | | 72.2 | 11.1 | F (3.102) | | 0.056 | 0.982 |  | |  | |  |
| Length (cm) | 36 | 164.5 | 5.8 | 23 | | 162.2 | 7.0 | 25 | | 163.2 | 7.1 | 23 | | 165.1 | 6.3 | F (3.103) | | 0.966 | 0.412 |  | |  | |  |
| BMI | 35 | 26.6 | 4.8 | 23 | | 27.8 | 4.6 | 25 | | 27.0 | 4.5 | 23 | | 26.5 | 4.0 | F (3.102) | | 0.460 | 0.711 |  | |  | |  |
| Pospartum systolic blood pressure | 30 | 111.4 | 11.5 | 22 | | 111.4 | 11.7 | 20 | | 112.2 | 10.5 | 16 | | 115.6 | 10.6 | F (3.84) | | 0.572 | 0.635 |  | |  | |  |
| Postpartum diastolic blood pressure | 30 | 68.1 | 6.3 | 22 | | 68.2 | 10.1 | 20 | | 67.2 | 7.7 | 16 | | 68.7 | 8.1 | F (3.84) | | 0.121 | 0.947 |  | |  | |  |
| Mother IL-6 (log10(pg/mL)) | 34 | 0.7 | 0.8 | 23 | | 0.9 | 0.8 | 25 | | 0.7 | 0.6 | 23 | | 0.6 | 0.6 | F (3.101) | | 0.731 | 0.536 |  | |  | |  |
| Mother IL-10 (log10(pg/mL)) | 34 | 1.5 | 1.0 | 23 | | 1.7 | 1.1 | 25 | | 1.5 | 0.8 | 23 | | 1.6 | 0.7 | F (3.101) | | 0.369 | 0.775 |  | |  | |  |
| Mother IL-6/IL-10 ratio (log10(pg/mL)) | 34 | -0.9 | 0.8 | 23 | | -0.8 | 0.9 | 25 | | -0.7 | 0.7 | 23 | | -1.0 | 0.7 | F (3.101) | | 0.415 | 0.743 |  | |  | |  |
| *Newborn* |  |  |  |  | |  |  |  | |  |  |  | |  |  |  | |  |  |  | |  | |  |
| Weight (g) | 36 | 3442.3 | 517.4 | 23 | | 3189.3 | 336.8 | 25 | | 3475.0 | 383.2 | 23 | | 3265.2 | 465.4 | F (3.103) | | 2.470 | 0.066 |  | |  | |  |
| Length (cm) | 35 | 50.7 | 1.9 | 23 | | 49.4 | 1.6 | 25 | | 50.9 | 1.6 | 23 | | 49.3 | 2.1 | F (3.102) | | 5.444 | 0.002 | 0.400 | | 1>4.2<3.3>4 * | |  |
| Newborn IL-6 (log10(pg/mL)) | 30 | 1.5 | 0.6 | 20 | | 1.7 | 0.6 | 20 | | 1.3 | 0.5 | 13 | | 1.5 | 0.6 | H (3) | | 3.368 | 0.338 |  | |  | |  |
| Newborn IL-10 (log10(pg/mL)) | 30 | 1.3 | 1.0 | 20 | | 1.7 | 0.9 | 20 | | 1.6 | 0.7 | 13 | | 1.3 | 0.8 | H (3) | | 3.027 | 0.388 |  | |  | |  |
| Newborn IL-6/IL-10 ratio (log10(pg/mL)) | 30 | 0.2 | 1.1 | 20 | | -0.1 | 0.9 | 20 | | -0.2 | 0.8 | 13 | | 0.2 | 0.8 | H (3) | | 2.399 | 0.494 |  | |  | |  |
| PDQ: Prenatal Distress Questionnaire; SRRS: Social Readjustment Rating Scale; CRP: Couple Relationship Quality Scale; STAI-E: State-Trait Anxiety Inventory; COS: Oviedo Sleep Questionnaire; PSS: Perceived Stress Scale; EDPS: Edinburgh Postnatal Depression Scale; BMI: Body Mass Index; ** p < 0.01; * p < 0.05; ns: not significant. | | | | | | | | | | | | | | | | | | | | | | | | |
