# Supplementary material for: Sex-specific vulnerabilities in early human neurodevelopment following SARS-CoV-2-induced maternal immune activation
Source: Eur Child Adolesc Psychiatry. 2025 Aug 27;35(1):239–51. doi: 10.1007/s00787-025-02837-z (PMC12916955; doi:10.1007/s00787-025-02837-z)
Supplement: Supplementary file 2 — (DOCX 46.7 KB) [file 787_2025_2837_MOESM2_ESM.docx]

| **Table S2.** Complete NBAS scores of COGESTCOV19 newborns at 6-week follow-up: sex-stratified case-control subdivision | | | | | | | | | | | | | | | | | | | |
| --- | --- | --- | --- | --- | --- | --- | --- | --- | --- | --- | --- | --- | --- | --- | --- | --- | --- | --- | --- |
|  | **Male Case** | | | **Female Case** | | | **Male Control** | | | | **Female Control** | | | **Adjusted for: Mother's age, gestational age,** **infant's age at NBAS assessment, years of education, child's weight and height, salary** | | | | | |
|  | **N= 32** | | | **N= 22** | | | **N= 24** | | | | **N= 22** | | |  |  |  |  |  |  |
|  | **n** | **Mean** | **SD** | **n** | **Mean** | **SD** | **n** | **Mean** | **SD** | **n** | | **Mean** | **SD** | **Statistical (*df*)** | **Value** | ***P*-value** | **Effect Size (η²)** | **Post-Hoc** |  |
| Habituation |  |  |  |  |  |  |  |  |  |  | |  |  |  |  |  |  |  |  |
| 1-Response decrement to light | 14 | 6.1 | 3.6 | 13 | 6.6 | 2.6 | 12 | 4.6 | 3.5 | 11 | | 5.9 | 2.0 | F (3. 46) | 1.384 | 0.262 |  |  |  |
| 2-Response decrement to rattle | 14 | 5.4 | 3.7 | 14 | 4.5 | 3.8 | 8 | 6.4 | 2.4 | 11 | | 3.4 | 2.4 | F (3. 43) | 0.970 | 0.418 |  |  |  |
| 3-Response decrement to bell | 9 | 6.8 | 3.5 | 9 | 5.4 | 3.9 | 7 | 3.0 | 1.4 | 10 | | 5.5 | 2.6 | F (3. 31) | 2.413 | 0.092 |  |  |  |
| 4-Response decrement to tactile stimulation of the foot | 5 | 4.8 | 3.6 | 3 | 7.9 | 2.6 | 2 | 4.4 | 0.9 | 3 | | 5.8 | 5.1 | F (3. 9) | 0.415 | 0.762 |  |  |  |
| Habituation selective composite score | 4 | 7.7 | 2.4 | 3 | 7.4 | 1.6 | 2 | 4.1 | 0.0 | 3 | | 6.6 | 2.2 | F (3. 8) | 0.646 | 0.698 |  |  |  |
| Habituation comprehensive composite score | 17 | 5.2 | 3.4 | 15 | 5.1 | 2.8 | 12 | 3.9 | 2.5 | 11 | | 4.8 | 1.9 | F (3. 51) | 0.861 | 0.468 |  |  |  |
| Orientation |  |  |  |  |  |  |  |  |  |  | |  |  |  |  |  |  |  |  |
| 5-Animate visual orientation | 30 | 5.5 | 2.2 | 20 | 3.8 | 2.7 | 20 | 5.4 | 2.5 | 22 | | 5.5 | 2.6 | F (3. 88) | 2.392 | 0.075 |  |  |  |
| 6-Animate visual and auditory orientation | 30 | 6.5 | 2.3 | 20 | 4.3 | 2.6 | 18 | 6.5 | 2.7 | 21 | | 6.5 | 2.6 | F (3. 85) | 4.132 | 0.009 | 0.137 | 1>2 ** |  |
| 7-Inanimate visual orientation | 29 | 4.1 | 2.4 | 20 | 2.2 | 2.1 | 18 | 3.9 | 2.9 | 19 | | 4.0 | 3.1 | F (3. 82) | 2.268 | 0.088 |  |  |  |
| 8-Inanimate visual and auditory orientation | 28 | 6.1 | 2.0 | 20 | 3.6 | 2.1 | 18 | 5.6 | 3.1 | 19 | | 5.7 | 2.8 | F (3. 81) | 4.675 | 0.005 | 0.159 | 1>2 ** |  |
| 9-Animate auditory orientation | 26 | 6.0 | 2.2 | 18 | 4.9 | 2.5 | 18 | 5.6 | 2.8 | 20 | | 5.7 | 2.3 | F (3. 78) | 0.963 | 0.415 |  |  |  |
| 10-Inanimate auditory orientation | 29 | 6.5 | 1.8 | 20 | 5.2 | 2.0 | 19 | 6.4 | 1.8 | 19 | | 5.3 | 2.1 | F (3. 83) | 4.108 | 0.009 | 0.140 | 1>2 1>4 * |  |
| 11-Alertness | 29 | 6.2 | 2.3 | 20 | 4.4 | 2.7 | 19 | 5.8 | 2.3 | 21 | | 5.7 | 2.6 | F (3. 85) | 2.683 | 0.052 | 0.094 | 1>2 * |  |
| Orientation selective composite score | 25 | 5.7 | 1.8 | 18 | 4.2 | 1.7 | 16 | 5.6 | 1.9 | 18 | | 5.7 | 2.2 | F (3. 73) | 3.130 | 0.031 | 0.125 | 1>2 * |  |
| Orientation comprehensive composite score | 30 | 5.9 | 1.7 | 20 | 4.0 | 1.7 | 20 | 5.5 | 1.9 | 22 | | 5.4 | 2.2 | F (3. 88) | 4.815 | 0.004 | 0.151 | 1>2 ** |  |
| Motor system |  |  |  |  |  |  |  |  |  |  | |  |  |  |  |  |  |  |  |
| 12-General tone | 32 | 8.6 | 2.0 | 22 | 8.8 | 1.5 | 24 | 8.3 | 1.9 | 22 | | 9.8 | 0.7 | F (3. 96) | 2.486 | 0.066 |  |  |  |
| 13-Motor maturity | 32 | 6.7 | 2.2 | 22 | 6.5 | 2.5 | 24 | 6.6 | 2.7 | 22 | | 6.9 | 2.7 | F (3. 96) | 0.181 | 0.909 |  |  |  |
| 14-Pull-to-sit | 32 | 5.3 | 1.7 | 22 | 5.3 | 2.3 | 22 | 5.4 | 1.7 | 22 | | 5.3 | 2.0 | F (3. 94) | 0.183 | 0.907 |  |  |  |
| 15-Defensive movement | 31 | 7.1 | 1.5 | 18 | 6.7 | 1.7 | 21 | 7.1 | 1.5 | 22 | | 6.6 | 1.9 | F (3. 88) | 1.075 | 0.364 |  |  |  |
| 16-Activity | 32 | 7.5 | 2.7 | 22 | 7.2 | 2.6 | 23 | 7.4 | 3.0 | 22 | | 7.8 | 2.6 | F (3. 95) | 0.296 | 0.828 |  |  |  |
| Motor system selective composite score | 31 | 7.0 | 1.3 | 18 | 6.9 | 1.2 | 20 | 7.2 | 1.0 | 22 | | 7.3 | 1.2 | F (3. 87) | 0.456 | 0.713 |  |  |  |
| Motor system comprehensive composite score | 32 | 7.0 | 1.3 | 22 | 6.9 | 1.2 | 24 | 6.9 | 1.3 | 22 | | 7.3 | 1.2 | F (3. 96) | 0.406 | 0.749 |  |  |  |
| State organization |  |  |  |  |  |  |  |  |  |  | |  |  |  |  |  |  |  |  |
| 17-Peak of excitement | 32 | 5.9 | 2.7 | 22 | 5.6 | 2.7 | 23 | 6.2 | 2.8 | 22 | | 4.7 | 3.1 | F (3. 95) | 2.220 | 0.091 |  |  |  |
| 18-Rapidity of build-up | 32 | 7.0 | 3.1 | 22 | 6.2 | 2.8 | 23 | 6.8 | 2.0 | 22 | | 7.1 | 2.4 | F (3. 95) | 0.691 | 0.560 |  |  |  |
| 19-Irritability | 32 | 5.6 | 2.5 | 20 | 5.3 | 2.5 | 22 | 5.3 | 2.8 | 22 | | 5.2 | 2.0 | F (3. 92) | 0.395 | 0.757 |  |  |  |
| 20-Lability of states | 32 | 6.6 | 2.8 | 22 | 6.5 | 2.1 | 23 | 6.0 | 2.6 | 22 | | 5.3 | 2.3 | F (3. 95) | 1.920 | 0.132 |  |  |  |
| State organization selective composite score | 32 | 6.3 | 2.2 | 20 | 6.2 | 1.9 | 21 | 6.3 | 1.8 | 22 | | 5.6 | 1.7 | F (3. 91) | 1.397 | 0.249 |  |  |  |
| State organization comprehensive composite score | 32 | 6.3 | 2.2 | 22 | 5.8 | 2.2 | 24 | 5.9 | 1.9 | 22 | | 5.6 | 1.7 | F (3. 96) | 1.602 | 0.194 |  |  |  |
| State regulation |  |  |  |  |  |  |  |  |  |  | |  |  |  |  |  |  |  |  |
| 21-Cuddliness | 31 | 4.7 | 1.9 | 21 | 4.5 | 1.6 | 23 | 4.7 | 1.9 | 22 | | 4.6 | 2.0 | F (3. 93) | 0.111 | 0.954 |  |  |  |
| 22-Consolability | 22 | 5.0 | 2.5 | 19 | 5.6 | 2.1 | 21 | 4.9 | 2.5 | 18 | | 5.2 | 3.4 | F (3. 76) | 0.120 | 0.948 |  |  |  |
| 23-Self-quieting | 26 | 4.6 | 3.2 | 22 | 4.7 | 3.1 | 22 | 3.9 | 2.9 | 19 | | 3.8 | 2.5 | F (3. 85) | 0.362 | 0.780 |  |  |  |
| 24-Hand-to-mouth | 32 | 2.4 | 3.0 | 22 | 4.5 | 2.6 | 24 | 3.2 | 2.8 | 22 | | 3.0 | 2.4 | F (3. 96) | 2.052 | 0.112 |  |  |  |
| State regulation selective composite score | 21 | 4.0 | 1.9 | 18 | 4.5 | 1.7 | 20 | 4.4 | 1.6 | 18 | | 4.1 | 1.9 | F (3. 73) | 0.343 | 0.795 |  |  |  |
| State regulation comprehensive composite score | 32 | 4.0 | 1.8 | 22 | 4.9 | 1.7 | 24 | 4.1 | 2.0 | 22 | | 4.1 | 1.8 | F (3. 96) | 0.961 | 0.415 |  |  |  |
| Autonomic stability |  |  |  |  |  |  |  |  |  |  | |  |  |  |  |  |  |  |  |
| 25-Tremolousness | 31 | 8.1 | 2.5 | 22 | 8.1 | 2.4 | 24 | 8.1 | 2.8 | 22 | | 9.0 | 1.9 | F (3. 95) | 0.361 | 0.782 |  |  |  |
| 26-Startles | 32 | 7.4 | 2.3 | 22 | 7.4 | 2.1 | 21 | 7.9 | 1.8 | 22 | | 7.0 | 2.3 | F (3. 93) | 0.671 | 0.572 |  |  |  |
| 27-Lability of skin | 32 | 7.7 | 2.5 | 22 | 7.4 | 2.8 | 24 | 7.9 | 2.4 | 22 | | 7.3 | 2.0 | F (3. 96) | 0.615 | 0.607 |  |  |  |
| Autonomic stability selective composite score | 30 | 6.6 | 1.4 | 22 | 6.0 | 1.3 | 21 | 6.5 | 1.1 | 22 | | 6.5 | 1.2 | F (3. 91) | 1.011 | 0.392 |  |  |  |
| Autonomic stability comprehensive composite score | 32 | 7.6 | 2.1 | 22 | 7.6 | 1.7 | 24 | 7.9 | 1.5 | 22 | | 7.8 | 1.5 | F (3. 96) | 0.341 | 0.796 |  |  |  |
| Smiles |  |  |  |  |  |  |  |  |  |  | |  |  |  |  |  |  |  |  |
| 28-Smiles | 30 | 2.7 | 2.0 | 22 | 1.3 | 1.3 | 24 | 1.4 | 1.8 | 22 | | 2.5 | 1.9 | F (3. 94) | 4.033 | 0.010 | 0.122 | ns |  |
| Suplementary items |  |  |  |  |  |  |  |  |  |  | |  |  |  |  |  |  |  |  |
| 29-Quality of alertness | 32 | 6.3 | 2.1 | 22 | 5.0 | 2.0 | 24 | 5.5 | 2.2 | 22 | | 6.3 | 2.3 | F (3. 96) | 2.639 | 0.054 | 0.082 | ns |  |
| 30-Cost of attention | 32 | 6.7 | 2.0 | 22 | 5.5 | 1.8 | 24 | 5.6 | 2.6 | 22 | | 5.5 | 2.0 | F (3. 96) | 4.521 | 0.005 | 0.132 | 1>4 **; 1>2 * |  |
| 31-Examiner facilitation | 32 | 6.2 | 2.4 | 22 | 5.3 | 1.5 | 24 | 5.3 | 2.6 | 22 | | 5.0 | 2.1 | F (3. 96) | 3.997 | 0.010 | 0.119 | 1>4 ** |  |
| 32-General irritability | 32 | 6.4 | 2.4 | 22 | 5.1 | 2.3 | 24 | 5.8 | 2.3 | 22 | | 5.7 | 2.0 | F (3. 96) | 2.118 | 0.104 |  |  |  |
| 33-Robustness and endurance | 32 | 6.8 | 2.3 | 22 | 6.0 | 2.0 | 24 | 5.8 | 2.6 | 22 | | 5.6 | 2.9 | F (3. 96) | 3.087 | 0.031 | 0.094 | 1>4 * |  |
| 34-State regulation | 32 | 7.8 | 1.4 | 22 | 6.5 | 2.1 | 24 | 6.8 | 2.4 | 22 | | 7.7 | 1.5 | F (3. 96) | 4.148 | 0.008 | 0.123 | 1>2 1>3 * |  |
| 35-Examiner´s emotional response | 32 | 7.9 | 2.0 | 22 | 7.4 | 2.0 | 24 | 8.3 | 1.9 | 22 | | 8.6 | 1.8 | F (3. 96) | 1.438 | 0.237 |  |  |  |
| Reflexes |  |  |  |  |  |  |  |  |  |  | |  |  |  |  |  |  |  |  |
| 1-Plantar grasp | 32 | 1.9 | 0.2 | 22 | 2.0 | 0.2 | 24 | 2.0 | 0.0 | 22 | | 2.0 | 0.0 | F (3. 96) | 0.794 | 0.500 |  |  |  |
| 2-Babinski | 32 | 1.9 | 0.2 | 22 | 2.0 | 0.2 | 24 | 2.0 | 0.0 | 22 | | 2.0 | 0.0 | F (3. 96) | 1.027 | 0.385 |  |  |  |
| 3-Ankle clonus | 31 | 1.5 | 0.5 | 20 | 1.4 | 0.5 | 23 | 1.5 | 0.5 | 20 | | 2.0 | 0.2 | F (3. 93) | 5.763 | 0.001 | 0.172 | 2<4 3<4 **; 1<4 * |  |
| 4-Rooting | 32 | 1.7 | 0.5 | 22 | 1.6 | 0.5 | 24 | 1.8 | 0.4 | 22 | | 1.9 | 0.3 | F (3. 96) | 1.435 | 0.238 |  |  |  |
| 5-Sucking | 32 | 1.9 | 0.3 | 22 | 2.0 | 0.2 | 24 | 1.9 | 0.3 | 22 | | 2.0 | 0.0 | F (3. 96) | 0.279 | 0.840 |  |  |  |
| 6-Glabella | 30 | 1.9 | 0.3 | 20 | 1.7 | 0.5 | 23 | 1.8 | 0.4 | 22 | | 2.0 | 0.2 | F (3. 93) | 2.214 | 0.093 |  |  |  |
| 7-Passive movements - legs | 32 | 1.8 | 0.4 | 22 | 1.9 | 0.4 | 23 | 1.9 | 0.3 | 22 | | 2.0 | 0.2 | F (3. 96) | 0.730 | 0.537 |  |  |  |
| 8-Passive movements - arms | 31 | 1.9 | 0.2 | 22 | 1.9 | 0.3 | 24 | 1.9 | 0.3 | 22 | | 1.9 | 0.3 | F (3. 96) | 0.177 | 0.912 |  |  |  |
| 9-Palmar grasp | 32 | 2.0 | 0.0 | 22 | 2.0 | 0.2 | 23 | 2.0 | 0.2 | 22 | | 2.0 | 0.0 | F (3. 96) | 0.816 | 0.489 |  |  |  |
| 10-Placing | 31 | 1.9 | 0.2 | 22 | 2.0 | 0.0 | 23 | 1.9 | 0.3 | 22 | | 2.0 | 0.0 | F (3. 96) | 0.619 | 0.604 |  |  |  |
| 11-Standing | 32 | 1.9 | 0.3 | 22 | 2.0 | 0.0 | 23 | 1.9 | 0.3 | 22 | | 2.0 | 0.0 | F (3. 96) | 0.950 | 0.420 |  |  |  |
| 12-Walking | 32 | 1.7 | 0.5 | 22 | 1.7 | 0.5 | 22 | 1.8 | 0.4 | 22 | | 1.9 | 0.3 | F (3. 96) | 0.529 | 0.664 |  |  |  |
| 13-Crawling | 31 | 1.8 | 0.4 | 21 | 1.7 | 0.5 | 22 | 1.9 | 0.3 | 22 | | 2.0 | 0.0 | F (3. 96) | 1.686 | 0.176 |  |  |  |
| 14-Incurvation (galiant response) | 31 | 1.8 | 0.4 | 22 | 1.6 | 0.5 | 21 | 1.7 | 0.5 | 22 | | 2.0 | 0.0 | F (3. 96) | 3.166 | 0.029 | 0.101 | 2<4 * |  |
| 15-Tonic deviation of head and eyes | 28 | 1.7 | 0.5 | 18 | 1.8 | 0.4 | 19 | 1.8 | 0.4 | 20 | | 1.6 | 0.5 | F (3. 93) | 1.893 | 0.138 |  |  |  |
| 16-Nystagmus | 27 | 1.5 | 0.5 | 17 | 1.4 | 0.5 | 18 | 1.7 | 2.3 | 18 | | 1.3 | 0.5 | F (3. 78) | 0.278 | 0.841 |  |  |  |
| 17-Tonic neck reflex | 30 | 1.7 | 0.5 | 19 | 1.8 | 0.4 | 21 | 1.6 | 0.5 | 19 | | 1.5 | 0.5 | F (3. 93) | 1.558 | 0.206 |  |  |  |
| 18-Moro reflex | 31 | 1.9 | 0.3 | 19 | 1.7 | 0.5 | 19 | 1.8 | 0.4 | 20 | | 1.9 | 0.4 | F (3. 96) | 1.026 | 0.386 |  |  |  |
| ** p < 0.01; * p < 0.05; ns: not significant. |  |  |  |  |  |  |  |  |  |  | |  |  |  |  |  |  |  |  |
